# Empathy motivation is preserved following amygdala damage

**DOI:** 10.1093/brain/awag074

**Published:** 2026-03-13

**Authors:** Julian A Scheffer, Justin Reber, C Daryl Cameron, Justin S Feinstein, Daniel Tranel

**Affiliations:** Department of Psychology, University of Western Ontario, London, ON N6A 5C2, Canada; Department of Neurology, University of Iowa, Iowa City, IA 52242, USA; Department of Neurology, University of Iowa, Iowa City, IA 52242, USA; Department of Psychology and Rock Ethics Institute, The Pennsylvania State University, University Park, PA 16802, USA; Department of Neurology, University of Iowa, Iowa City, IA 52242, USA; Department of Neurology, University of Iowa, Iowa City, IA 52242, USA

**Keywords:** experience sharing, perspective taking, decision-making, free choice, mental effort, amygdala lesions

## Abstract

Damage to the amygdala has been linked to impairments in empathy, typically documented as deficits in accurately identifying others’ emotional experiences, especially fear. This has led some to theorize that amygdala dysfunction is a core feature of psychopathy. There is growing evidence, however, that motivation to empathize is distinct from empathic accuracy. Moreover, anecdotal observations in patients with amygdala lesions have noted their tendencies to approach, rather than avoid, empathic encounters with strangers, even when the patients have impairments in empathic accuracy.

We conducted a novel investigation specifically examining empathy motivation in patients with amygdala damage. We used a free-choice paradigm to assess motivation to empathize.

We found that damage to the amygdala was not associated with avoidance of affective or cognitive forms of empathy motivation. Patients with amygdala lesions (*n* = 21) exhibited similar levels of empathy motivation compared with patients with damage outside the amygdala (*n* = 22) and healthy individuals with no brain damage (*n* = 24).

These findings suggest that amygdala damage does not necessarily disrupt the motivation to empathize. A potential implication of the findings is that amygdala damage or dysfunction may not be associated with traits such as callousness, apathy or lack of caring that are often linked to psychopathy.

## Teaser

Damage to the amygdala is associated with reduced empathic accuracy. However, Scheffer *et al*. show that patients with amygdala lesions are as motivated to empathize as those with other brain lesions or healthy controls, indicating that motivational components of empathy are preserved.

**See Zhao (https://doi.org/10.1093/brain/awag110) for a scientific commentary on this article.**

## Introduction

Converging evidence from neuroimaging and neuropsychological research supports the conclusion that the amygdala is involved in empathy, here defined as identifying, sharing and understanding other people’s emotional experiences. Functional neuroimaging has found that amygdala activation occurs when participants simulate other people’s pain^1^ or recognize fear in other people,^2-4^ which often catalyses prosocial concern or behaviour. Fittingly, extraordinary altruistic behaviour (e.g. kidney donation to strangers) has been correlated with larger amygdalae,^4^ while reduced activity and grey matter in the amygdala have been correlated with callous, antisocial or psychopathic traits.^5,6^ Neuropsychological approaches have shown that patients with focal amygdala damage have difficulty identifying fear in other people’s facial expressions,^7,8^ written statements^9^ and in musical cues.^10,11^ Based on this evidence, amygdala dysfunction or damage (particularly during early childhood) has been theorized to contribute to socioemotional impairments as well as to the development of callous, sociopathic or psychopathic traits.^9,12,13^

However, some patients with amygdala lesions are also exceptionally altruistic.^14-16^ Patient SM, a woman with bilateral amygdala calcification due to Urbach–Wiethe disease, has profound empathic concern for others and a history of hyper-altruistic behaviour,^15^ such as giving her only winter coat to a freezing stranger (while she herself lived in poverty) and walking several miles in a severe thunderstorm to comfort a friend.^14^ Additionally, patients with amygdala lesions approach fear-inducing situations^7^ and complete strangers more than people without damage to these regions.^17,18^ As a result, patients with amygdala lesions could have more motivation to empathize with strangers.

This apparent contradiction in this research highlights a critical gap in the study of empathy—much of the extant literature conflates motivation to empathize with ability to empathize.^19^ When deciding whether to approach or avoid empathic encounters, there is growing evidence that people weigh its anticipated rewards (e.g. trust and approval) against its costs (e.g. financial or emotional exhaustion).^19-21^ The amygdala may be involved in this process, as it was recently reported that individuals with basolateral amygdala lesions are not as discerning about who to be prosocial towards depending on the nature of their relationship with the target (e.g. from best friends to complete strangers).^22^ Although some studies have examined amygdala morphology and its relation to altruistic behaviour in neurologically healthy individuals,^4,6^ our focus on amygdala damage provides a unique vantage point on the necessity of the region for empathy and any associated prosocial behaviour. Lesion studies offer a clinically valuable and naturalistic opportunity to observe affective, behavioural or cognitive differences in patients who have sustained brain damage due to trauma, ischaemia or surgical procedures. To our knowledge, no research to date has examined whether the altered empathic behaviour of patients with amygdala lesions may be due to differences in motivation to empathize.

Here, we used a free-choice measure of empathy motivation, the Empathy Selection Task (EST),^23^ in which participants make decisions to either approach or avoid empathy in two variants—one designed to target experience sharing (i.e. affective empathy) and the other to target perspective taking (i.e. cognitive empathy). Binary selection tasks are used extensively to study motivation and individual variation in mental effort avoidance,^24-26^ including in clinical samples (e.g. schizophrenia).^27^ The EST was adapted from such work, and has been used to uncover robust patterns of empathy avoidance in neurologically healthy participants (particularly, when they find it mentally effortful),^23,28^ including their preferences to empathize with loved ones compared with strangers.^23,28^ The EST can also reliably quantify individual variation in empathy motivation and correlates with multi-component dispositional trait empathy using well-established self-report measures [e.g. the Interpersonal Reactivity Index (IRI)].^23,29-31^ Thus, the EST provides a compelling measure to assess empathy motivation in patients with amygdala lesions. We opted to use two variants because deficits in each type of empathy have been documented elsewhere in amygdala lesion patients.^32,33^ We pre-registered our methods, hypothesis and analysis plan during data collection (https://osf.io/rzva5). However, for the current study, for simplicity and specificity, we used the lesion comparison method for patients with amygdala lesions only (Supplementary Table 1).

Patients with amygdala lesions were compared against two other groups: (i) patients with brain damage outside of the amygdala [brain-damaged comparison (BDC)]; and (ii) demographically matched healthy comparison participants (HC). Our central focus was on comparing the patients with amygdala lesions against brain-damaged comparison and healthy comparison groups (noted from here onward as ‘comparison groups’), with case-matching conducted based on the demographics of the amygdala lesion group.^34,35^

We hypothesized that patients with amygdala lesions would exhibit significantly higher motivation to empathize in relation to the comparison groups given documented evidence that these patients may be predisposed for greater empathic responding and behaviours (H1). We additionally examined the extent of task engagement on the affective EST and cognitive EST to determine how these might affect the main findings. We also hypothesized that patients with amygdala lesions would perceive less mental effort associated with empathy compared with the comparison groups, given evidence that people prefer to avoid cognitive effort which tracks with their avoidance of empathy^24,36,37^ (H2) but that patients with amygdala lesions may not perceive these same costs (e.g. their fearlessness and tendencies to approach and help strangers might suggest less mental effort or aversion to empathy-evoking experiences). Moreover, that their reduced perceptions of mental effort with empathy should track with increased empathy choice proportion. We report additional hypotheses and analyses in the Supplementary material.

## Materials and methods

### Participants

We recruited 21 patients with unilateral amygdala lesions (13 left, 8 right), 22 patients with lesions outside of the amygdala and ventromedial prefrontal cortex (BDC; 10 left, 8 right, 4 bilateral) and 24 demographically matched healthy comparison (HC) participants (see Supplementary Table 1 for an explanation of participant selection). Demographic information is presented in Table 1. All patients had detailed neuropsychological testing and structural neuroimaging performed in the chronic epoch (>3 months post-lesion). Patients were screened to exclude individuals with a history of learning disabilities, psychiatric disorders, substance abuse, premorbid personality disorders, developmental epilepsy and other neurological conditions not related to their specified lesion.^38^

**Table 1:** Participant demographics

Participant group	Age, mean (SD)	Sex	Years of education, mean (SD)	Laterality	Chronicity years, mean (SD)
Amygdala lesions	48.09 (10.76)	8 male, 13 female	15.14 (2.56)	13 left, 8 right	11.03 (7.21)
Brain-damaged comparison	47.70 (13.30)	9 male, 13 female	14.64 (2.74)	10 left, 8 right, 4 bilateral	7.66 (8.91)
Healthy comparison	51.82 (12.38)	9 male, 15 female	16.75 (2.27	NA	NA
Group tests	*F*(2,64) = 0.80, *P =* 0.452, $\eta_{p}^{2}$ = 0.02	χ^2^ = 0.06, *P =* 0.969	*F*(2,64) = 4.44, *P =* 0.016, $\eta_{p}^{2}$ = 0.12	χ^2^ = 73.81, *P* < 0.001	*F*(1,41) = 1.84, *P =* 0.182, $\eta_{p}^{2}$ = 0.04

Case-matching was prioritized for age, then sex, then years of education for the amygdala lesions group with individuals in the brain-damaged comparison and healthy comparison groups. NA = not applicable; SD = standard deviation.

Participants were excluded from analyses conducted within each EST (affective, cognitive) using pairwise deletion approaches if they were missing at least one choice response within the given task (i.e. missing a response for their deck selection). In the affective EST, we had to exclude one amygdala lesion patient and two BDC patients for missing choice responses in this task. We further observed that one healthy comparison participant left three blank written responses for this EST, but we opted to retain their task data given this was minimal (<7.5%). In the cognitive EST, we had to exclude two amygdala lesion patients for missing choice responses in this task. We also observed that two amygdala lesion patients (one to two trials) had missing valence ratings, but we opted to retain their task data given this was minimal missing data (≤ 5%). All procedures were approved by the Institutional Review Board and are in accordance with the Declaration of Helsinki.

### Neuroanatomical analysis

**Figure 1:**
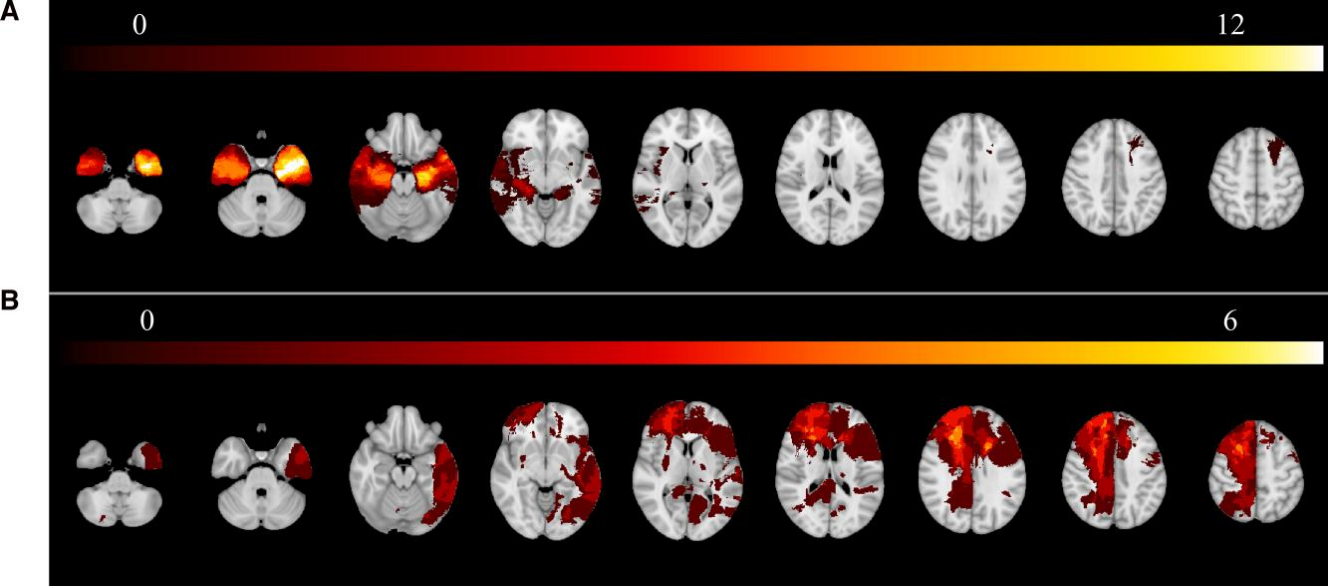
**Lesion overlap maps of (A) amygdala lesioned and (B) brain-damaged comparison patients.** Yellow regions reflect greater lesion overlap in the sample; 21 patients with unilateral amygdala lesions (13 left, 8 right), 22 patients with lesions outside of the amygdala (brain-damaged comparisons, or BDCs; 10 left, 8 right, 4 bilateral).

Lesion boundaries were segmented manually for all patients’ scans according to standard procedures.^39,40^ Prior to 2006, lesion volumes were traced directly onto a template brain using the subjects’ scans using Brainvox and MAP-3 new objective templating techniques.^41^ Lesions traced since 2006 were manually traced directly on the native T1-weighted scans using FSL^42^ and then transformed to MNI152 (Montreal Neurological Institute) space. A neurologist blinded to cognitive data and the study hypotheses reviewed and corrected the anatomical accuracy of each lesion mask in both native and MNI space (Fig. 1).

### Procedure and materials

Participants completed a set of tasks that were programmed into an online Qualtrics survey. The researchers provided participants with a laptop computer with a keyboard and mouse that was used to input responses. A portion of the individual difference measures were completed on written forms in-person or in a take-home packet.

#### Affective EST

**Figure 2:**
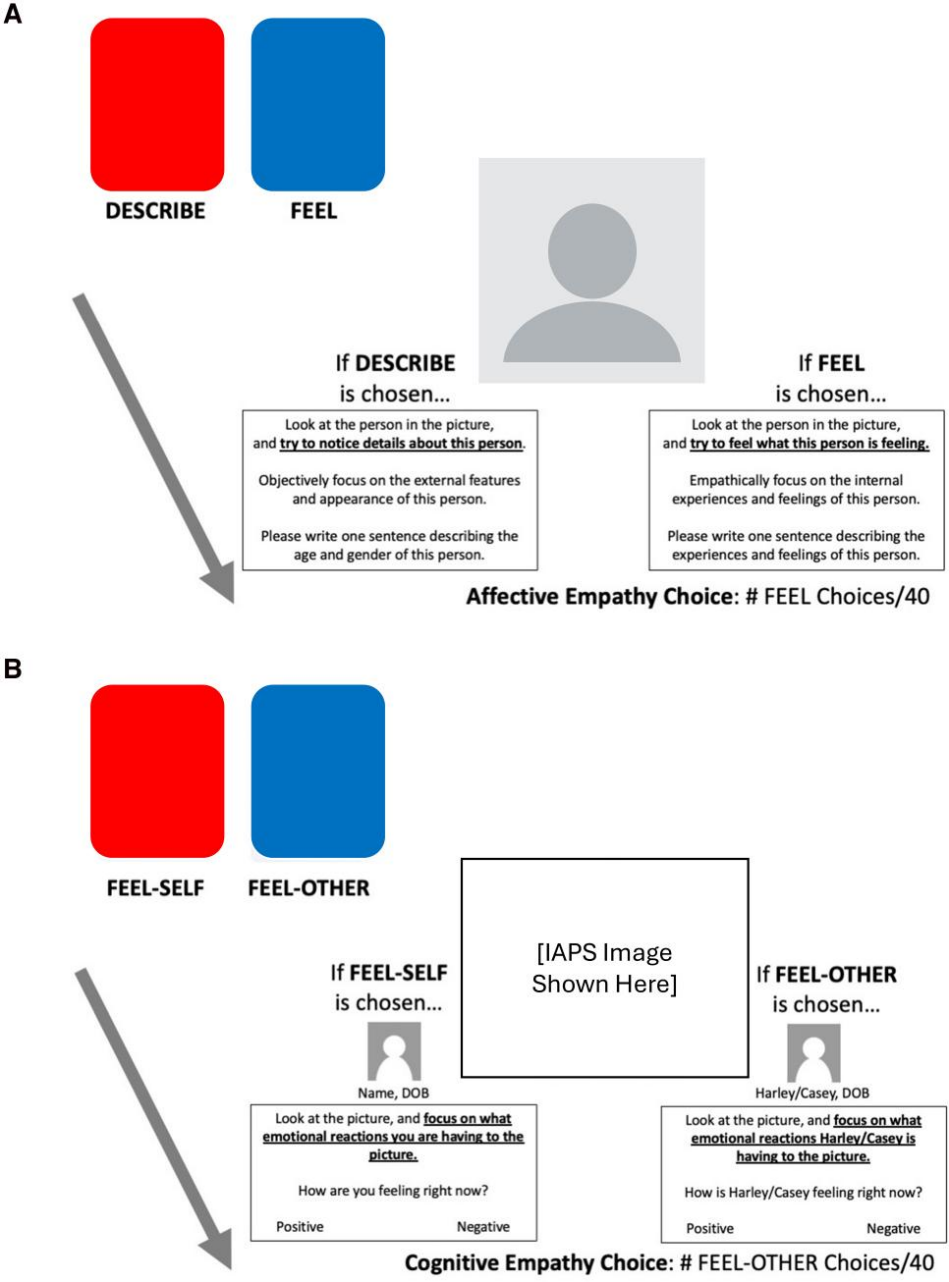
**Affective (A) and cognitive (B) Empathy Selection Task (EST) schematics.** In the Cognitive EST (**B**), name, date of birth (DOB) on the FEEL-SELF deck refers to the participant’s piped-in information. These are schematics of the task for a rough visualization only. IAPS = International Affective Picture System.

Participants completed 40 trials of an affective variant of the EST to assess preferences to share in the experiences of other people.^23^ Participants chose between a blue-coloured ‘FEEL’ deck (empathy choice) which instructed participants to focus on internal experiences and feelings of the person in the image, or a red-coloured ‘DESCRIBE’ deck (non-empathy choice) which instructed participants to focus on external features and appearances of the person in the image. After making a choice on a trial, they were shown one of 40 randomly selected images of a child with instructions from their chosen deck. Participants could not submit their written response until at least 10 s had elapsed (Fig. 2A).

#### Cognitive EST

Participants completed 40 trials of a cognitive variant of the EST to assess preferences to imagine the emotional responses of another person.^23^ Participants chose between a blue-coloured ‘FEEL-OTHER’ (empathy choice) which instructed them to concentrate on the emotional reaction that the other person was having, or a red-coloured ‘FEEL-SELF’ (non-empathy choice) which instructed them to concentrate on the emotional reaction that they themselves were having. Before participants began the task, they entered their first name as well as the month and date of their birth (e.g. ‘Larry, October 30’). Participants were also randomly assigned to be paired with either Harley (October 3) or Casey (January 14) as the FEEL-OTHER deck participant. On each trial, after participants made their choice, a randomly selected medium-arousal image from the International Affective Picture System (IAPS) database was displayed and participants reported on whether they themselves (FEEL-SELF choice) were feeling positive or negative, or whether the other person (FEEL-OTHER choice) was feeling positive or negative. Participants could not submit their response until at least 10 s had elapsed (Fig. 2B).

#### Post-task questionnaires

Participants provided responses to the following questions: ‘What was it like performing the task?’, ‘Did you develop a preference for one of the decks?’ and ‘How did you choose between the decks?’^23,24^

#### NASA Task Load Index

Participants answered the following NASA Task Load Index^43^ questions in response to both decks within each task: ‘How mentally demanding was this deck?’, ‘How hard did you have to work to accomplish your level of performance with this deck?’, ‘How insecure, discouraged, irritated, stressed and annoyed were you by this deck?’ and ‘How successful were you in accomplishing what you were asked to do in this deck?’ The first two questions were combined to indicate perceived effort, the third question indicated negative affect, and the fourth indicated perceived efficacy/success for engaging and achieving the deck’s instructions.^23,37^

#### Individual differences

After completing the aforementioned tasks, participants completed individual difference measures: demographics (e.g. gender, ethnicity, age), the IRI^44^ to assess participant endorsement of facets of (dispositional) empathy, the Self-Reported Altruism Scale^45^ to assess self-reports of altruistic behaviour, and the Behavioural Inhibition and Behavioural Activation System Scales^46^ to assess approach or avoidance biases in participants given prior work on amygdala damage and approach bias. Participants completed the other written self-report measures either in person or were given a take-home packet that included the Personality Inventory for the Diagnostic and Statistical Manual of Mental Disorders (DSM-5) self and informant report (PID-5, PID-5-IRF),^47,48^ and the Psychopathic Personality Inventory-Revised (PPI-R)^49^ to complete and mail in using a prepaid envelope. The informant was instructed to be a spouse, family member, roommate or close friend who knew them to fill out the packet. Several participants did not return the take-home assessments, and many did not include one of the questionnaires when returning the packet.

## Results

### Empathy motivation

To assess empathy motivation, we examined the proportion of choosing empathy: number of FEEL choices out of 40 total choices in the affective EST; and number of FEEL-OTHER choices out of 40 total choices in the cognitive EST. We report null-hypothesis significance test (NHST) ANOVA and separate Bayesian ANOVAs to determine the magnitude of the group difference effect size, 95% confidence region and report the region of practical equivalence (ROPE) using the bayesanova package in R (i.e. whether the magnitude of the effect size was no effect, small, medium or large and the highest percentage of support for the size of the effect).^50,51^

**Figure 3:**
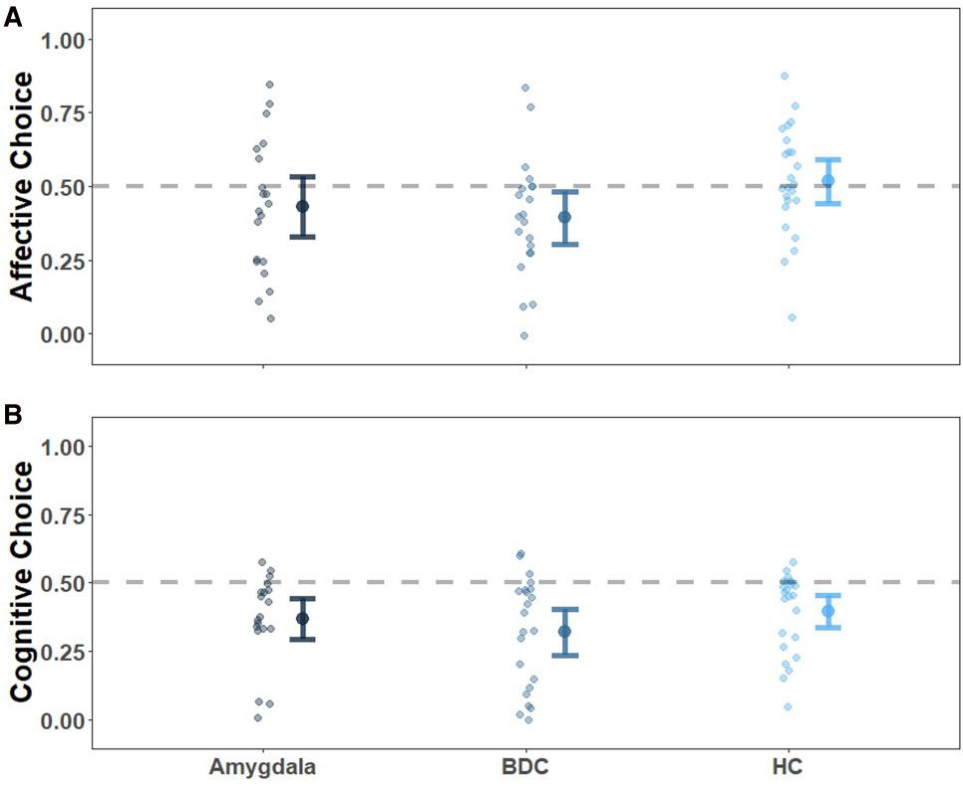
**Choice proportion in the affective (A) and cognitive (B) Empathy Selection Task.** BDC refers to the brain-damaged comparison group, and HC refers to the healthy comparison group. No group effects were observed between patients with amygdala lesions and the BDC and HC groups (affective EST posterior mean: 0.05, −0.13; 100% posterior probability of no effect; cognitive EST posterior mean: 0.07, −0.04; 100% posterior probability of no effect). The dashed grey line reflects chance proportion (0.50). Individual participant scores are jittered slightly in position to prevent overlap beside the group means (larger dots) and 95% confidence intervals (error bars).

Across the entire sample, participants did not significantly avoid the empathy deck (Fig. 3A) in the affective EST [FEEL choice: mean = 0.45, SD = 0.21, *t*(64) = −1.92, *P* = 0.059, 95% confidence interval (CI) of the mean difference (−0.10, 0.00)]. In the cognitive EST, participants avoided the empathy deck [FEEL-OTHER choice: mean = 0.36, SD = 0.17, *t*(64) = −6.47, *P* = <0.001, 95% CI of the mean difference (−18, −0.09); Fig. 3B].

#### Empathy motivation appears preserved in patients with amygdala lesions

Based on the NHST, we did not find support for H1 (patients with amygdala lesions will choose empathy more compared with the comparison groups) in the affective EST [*F*(2,62) = 2.16, *P* = 0.123, $\eta_{p}^{2}$ = 0.07] or in the cognitive EST [*F*(2,62) = 1.16, *P* = 0.319, $\eta_{p}^{2}$ = 0.04]. The two EST variants showed no statistically significant correlation [*r* = 0.01, *P* = 0.945, 95% CI (−0.24, 0.25)], which suggests that performance on these tasks may be interpreted separately (Table 2 and Fig. 3).

**Table 2:** Empathy deck choice proportion in the Empathy Selection Tasks

Participant group	Empathy choice, mean (SD)	95% CI of the mean difference	*t*	*P*	*n*	Cohen’s *d*
**Affective EST**						
Amygdala lesions	0.43 (0.23)	[−0.18, 0.04]	−1.36	0.190	20	−0.30
Brain-damaged comparison	0.39 (0.20)	[−0.20, −0.01]	−2.42	0.025	21	−0.53
Healthy comparison	0.52 (0.19)	[−0.06, 0.10]	0.44	0.664	24	0.09
**Cognitive EST**						
Amygdala lesions	0.37 (0.17)	[−0.21, −0.05]	−3.47	0.003	19	−0.80
Brain-damaged comparison	0.32 (0.20)	[−0.27, −0.09]	−4.24	<0.001	22	−0.90
Healthy comparison	0.40 (0.15)	[−0.16, −0.04]	−3.46	0.002	24	−0.71

Participants were excluded for not completing all choice trials in the affective Empathy Selection Task (EST) (*n* = 1 amygdala lesion patient; *n* = 2 brain-damaged comparison patients) and cognitive EST (*n* = 2 amygdala lesion patients).

#### Bayesian comparisons reveal no support for differences in empathy motivation

For the affective EST, no effect was observed between patients with amygdala lesions and brain-damaged comparison patients [posterior mean = 0.05, 95% CI (−0.00, 0.10); 100% posterior probability inside ROPE of no effect] and between patients with amygdala lesions and healthy comparison participants [posterior mean = −0.13, 95% CI (−0.17, −0.07); 100% posterior probability inside ROPE of no effect].

For the cognitive EST, there were violations of normality with preferences for avoiding empathy. Therefore, we report and interpret these Bayesian comparisons with caution. No effect was observed between patients with amygdala lesions against brain-damaged comparison patients [posterior mean = 0.07, 95% CI (0.02, 0.12); 100% posterior probability inside ROPE of no effect] and between patients with amygdala lesions and healthy comparison participants [posterior mean = −0.04, 95% CI (−0.09, 0.01); 100% probability inside ROPE of no effect].

These findings support the null hypothesis that patients with amygdala lesions do not exhibit significant differences in their motivation to engage in either affective or cognitive empathy, compared with patients with lesions outside the amygdala and compared with individuals with no brain lesions.

### Affective EST engagement

The affective EST presents the images of children drawn from previous research.^23^ Many of these children appear to be expressing negatively valenced emotions (e.g. sadness, fear),^52^ therefore, we coded whether participants wrote in more negative or positive affect and emotion words within their responses by comparing these responses against emotion dictionaries,^53^ and broke this down by their participant group to determine whether any groups may have unexpectedly reported more or less negative and positive emotion words (Table 3). After initially identifying words that overlapped with the emotion dictionaries, a coding team that was blinded to the participant groups, deck choices and research hypotheses identified any wrongly identified emotion words (i.e. ‘like’, ‘play’, ‘please’, ‘pretty’) in the context of the written responses (e.g. ‘This toddler looks like they are either scared or surprised’). These words generally did not indicate the identification of an emotion or feeling state in the target of the image. We also created a combined emotion word dictionary that included positive and negative emotion words from these dictionaries^53^ that additionally included emotion words identified by the coding team to be missing from the dictionaries (e.g. ‘somber’, ‘curious’, ‘astonished’). This was done to determine whether any findings obtained using the original emotion word dictionaries would replicate when also incorporating missing emotion and feeling state words that were identified by the coding team.

**Table 3:** Affective Empathy Selection Task (EST) emotion word use proportion

	Amygdala lesions, mean (SD)	Brain-damaged comparison, mean (SD)	Healthy comparison, mean (SD)
**FEEL**			
Positive	0.10 (0.11)	0.11 (0.10)	0.16 (0.10)
Negative	0.25 (0.15)	0.21 (0.10)	0.29 (0.11)
**DESCRIBE**			
Positive	0.04 (0.09)	0.05 (0.07)	0.04 (0.07)
Negative	0.08 (0.18)	0.06 (0.09)	0.07 (0.11)

Proportion of trials in which participants used a positive or negative emotion word out of the total number of trials (40).

In exploratory analyses, we conducted a series of binomial generalized linear mixed models to examine whether the use of positive or negative emotion words would vary based on deck choice (FEEL/empathy, DESCRIBE/non-empathy) and participant group. We would expect that more emotion words would be used on the empathy deck compared with non-empathy deck in this task, but we had no prior predictions about the participant groups varying in their emotion word usage.

#### Patients with amygdala lesions did not differ in how they identified emotions in the affective EST

For use of positive emotion words, participants overall were more likely to use a positive emotion word on the empathy compared with non-empathy deck [b = 1.09, standard error (SE)(b) = 0.34, *P* = 0.001], but there were no differences across participant groups [b = 0.02, SE(b) = 0.13, *P* = 0.900] and no interaction of deck choice and participant group [b = 0.17, SE = 0.11, *P* = 0.125].

For use of negative emotion words, participants overall were more likely to use a negative emotion word on the empathy compared with the non-empathy deck [b = 3.04, SE(b) = 0.32, *P* < 0.001], but there were no differences across participant groups [b = 0.08, SE(b) = 0.12, *P* = 0.484] or an interaction of deck choice and participant group [b = −0.16, SE(b) = 0.10, *P* = 0.121].

The combined emotion word dictionary showed a similar pattern. Participants overall were more likely to use an emotion word on the empathy compared with non-empathy deck [b = 4.40, SE(b) = 0.39, *P* < 0.001], but there were no differences across participant groups [b = 0.12, SE(b) = 0.15, *P* = 0.430] or an interaction of deck choice and participant group ([b = −0.07, SE(b) = 0.13, *P* = 0.593].

In summary, we did not obtain sufficient evidence that patients with amygdala lesions completed the affective EST instructions differently than comparison groups. However, we observed an effect of increased likelihood of using an emotion word when choosing the empathy deck compared with non-empathy deck.

### Cognitive EST engagement

We explored whether participants recognized that the other person would either feel more positive or more negative in responses to the emotional images. The images selected from the IAPS database have normed valence ratings provided^54^ and 20 images were more positive and 20 images were more negative. Therefore, we examined how the valence ratings matched what participants identified in other people as well as in themselves based on the FEEL-OTHER and FEEL-SELF deck choices, respectively.

#### Patients with amygdala lesions did not differ in attributions of feelings in the cognitive EST

When choosing the empathy deck for the more positive images, there were no significant group differences in the proportion of rating the other person as feeling more positive [*F*(2,59) = 0.20, *P* = 0.819, $\eta_{p}^{2}$ = 0.01]. When choosing the empathy deck for more negative images, there again were no significant group differences in the proportion of rating the other person as feeling more negative [*F*(2,58) = 2.43, *P* = 0.097, $\eta_{p}^{2}$ = 0.08].

When choosing the non-empathy deck for more positive images, there were no group differences in the proportion of reporting feeling more positive [*F*(2,62) = 0.23, *P* = 0.795, $\eta_{p}^{2}$ = 0.01]. When choosing the non-empathy deck for more negative images, again there were no group differences in the proportion of reporting feeling more negative [*F*(2,62) = 0.23, *P* = 0.794, $\eta_{p}^{2}$ = 0.01].

In summary, the findings do not indicate that patients with amygdala lesions completed the cognitive EST instructions differently than comparison groups.

### Empathy’s cognitive cost ratings

**Figure 4:**
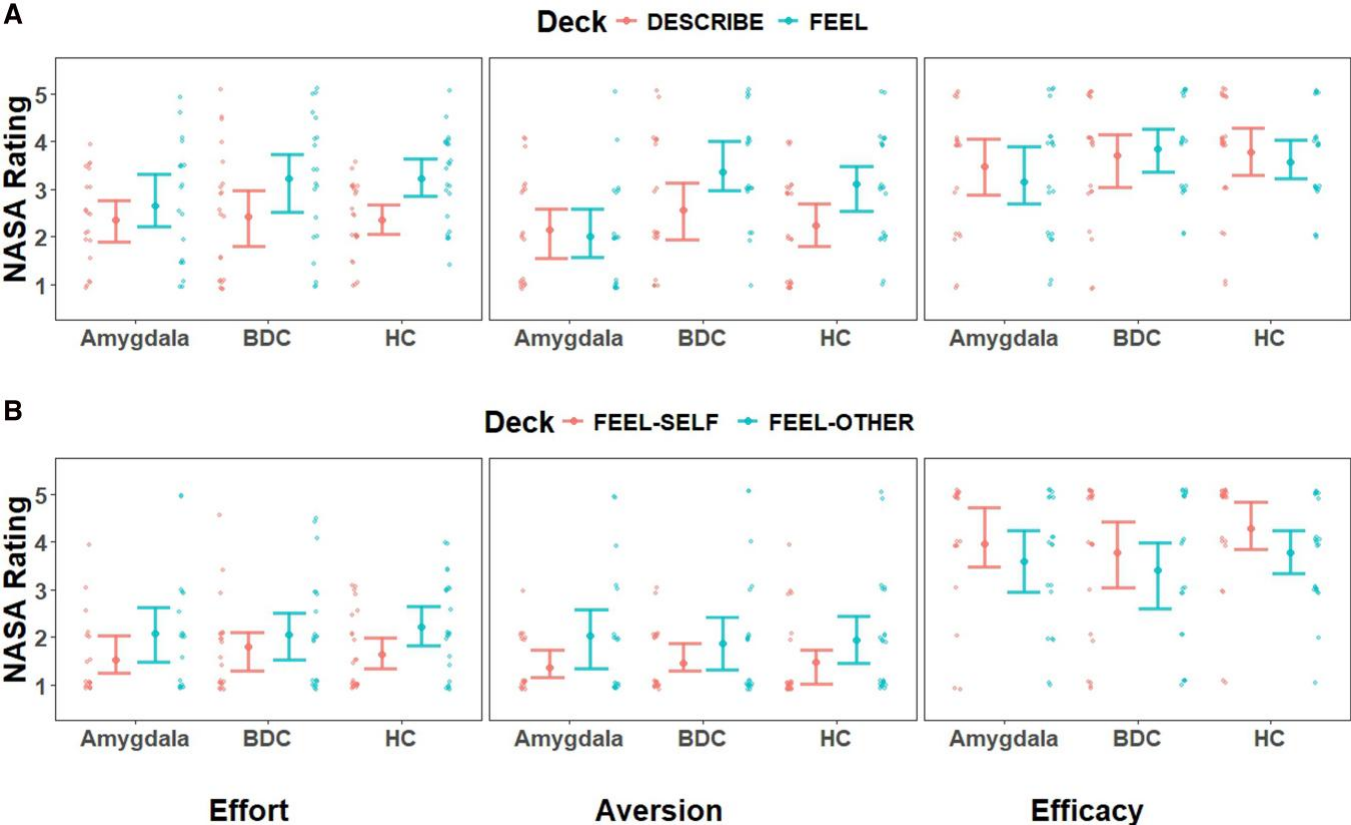
**Self-reports of cognitive costs in the (A) affective and (B) cognitive Empathy Selection Task.** BDC refers to the brain-damaged comparison group, and HC refers to the healthy comparison group. Individual participant scores are jittered slightly in position to prevent overlap beside the group means (larger dots) and 95% confidence intervals (error bars).

To assess perceptions of cognitive costs with empathy and non-empathy decks, we examined the ratings on the NASA Task Load Index^43^ for effort, aversiveness and efficacy for each deck collapsed across the three groups using NHST (Table 4 and Fig. 4). We also adopted Bayesian comparisons to provide evidence towards the null or alternative hypothesis (Supplementary Table 2).

**Table 4:** Empathy Selection Task deck ratings on the NASA Task Load Index

		Affective EST						
Participant group	FEEL deck, mean (SD)	DESCRIBE deck, mean (SD)	Mean difference [95% CI]	*P*	Cohen’s *d*	Choice *r*^a^	Choice *P*	*n*
**Effort**								
Amygdala lesions	2.68 (1.25)	2.38 (0.96)	[−0.19, 0.79]	0.214	0.29	0.12	0.625	20
Brain-damaged comparison	3.15 (1.38)	2.50 (1.34)	[0.13, 1.17]	0.017	0.58	−0.33	0.153	20
Healthy comparison	3.23 (0.94)	2.35 (0.77)	[0.33, 1.42]	0.003	0.68	−0.09	0.686	24
**Aversion**								
Amygdala lesions	2.05 (1.14)	2.15 (1.14)	[−0.65, 0.45]	0.705	0.09	−0.09	0.718	20
Brain-damaged comparison	3.40 (1.14)	2.70 (1.34)	[0.09, 1.31]	0.027	0.54	−0.55	0.013	20
Healthy comparison	3.08 (1.13)	2.21 (1.10)	[0.44, 1.31]	<0.001	0.85	0.03	0.895	24
**Efficacy**								
Amygdala lesions	3.25 (1.33)	3.40 (1.31)	[−0.66, 0.36]	0.545	−0.14	0.43	0.060	20
Brain-damaged comparison	3.80 (1.01)	3.65 (1.31)	[−0.55, 0.85]	0.659	0.10	0.00	0.992	20
Healthy comparison	3.63 (0.97)	3.75 (1.22)	[−0.74, 0.49]	0.678	−0.09	0.25	0.231	24

		Cognitive EST						
Participant group	Feel-Other deck, mean (SD)	Feel-Self deck, mean (SD)	Mean difference [95% CI]	*P*	Cohen’s *d*	Choice *r*^a^	Choice *P*	*n*
**Effort**								
Amygdala lesions	2.11 (1.25)	1.55 (0.85)	[−0.64, 1.17]	0.076	0.43	−0.06	0.805	19
Brain-damaged comparison	2.07 (1.17)	1.73 (0.94)	[−0.02, 0.70]	0.061	0.42	0.01	0.977	22
Healthy comparison	2.23 (1.00)	1.65 (0.79)	[0.15, 1.02]	0.011	0.57	−0.40	0.053	24
**Aversion**								
Amygdala lesions	2.05 (1.35)	1.42 (0.61)	[−0.02, 1.28]	0.055	0.47	−0.11	0.649	19
Brain-damaged comparison	1.95 (1.29)	1.50 (0.68)	[−0.09, 1.00]	0.096	0.37	−0.44	0.042	22
Healthy comparison	1.92 (1.21)	1.46 (0.88)	[0.06, 0.85]	0.024	0.49	−0.24	0.249	24
**Efficacy**								
Amygdala lesions	3.53 (1.39)	4.05 (1.35)	[−1.38, 0.33]	0.213	−0.30	0.48	0.040	19
Brain-damaged comparison	3.32 (1.62)	3.73 (1.61)	[−0.81, −0.01]	0.047	−0.45	0.22	0.342	22
Healthy comparison	3.75 (1.07)	4.33 (1.20)	[−1.10, −0.07]	0.027	−0.48	0.33	0.112	24

In the Affective Empathy Selection Task (EST), participants in the amygdala group (*n* = 1) and brain-damaged comparison group (*n* = 2) did not respond to the NASA Task Load Index. In the Cognitive EST, participants in the amygdala group (*n* = 2) did not respond to the NASA Task Load Index.

^a^The association between empathy choice and cognitive cost difference ratings (e.g. Effort_empathy_ − Effort_non-empathy_ = EffortDifferenceRating_effort_).

In the affective EST, participants reported the empathy deck more effortful [*F*(1,63) = 18.42, *P* < 0.001, $\eta_{p}^{2}$ = 0.23], more aversive [*F*(1,63) = 11.41, *P* = 0.001, $\eta_{p}^{2}$ = 0.15], but that it did not differ on efficacy compared with the non-empathy deck [*F*(1,63) = 0.08, *P* = 0.782, $\eta_{p}^{2}$ = 0.00]. In the cognitive EST, participants reported the empathy deck more effortful [*F*(1,64) = 14.77, *P* < 0.001, $\eta_{p}^{2}$ = 0.19], more aversive [*F*(1,64) = 12.73, *P* < 0.001, $\eta_{p}^{2}$ = 0.17] and reported less efficacy [*F*(1,64) = 9.91, *P* = 0.001, $\eta_{p}^{2}$ = 0.13]. However, we did not find consistent support for H2 (that compared with the comparison groups, patients with focal amygdala lesions ratings of cognitive costs would be less for the empathy deck compared with the non-empathy deck in the two ESTs).

#### Empathy felt less aversive to patients with amygdala lesions

In the affective EST, there was no significant Group × Deck interaction on effort [*F*(2,61) = 1.35, *P* = 0.267, $\eta_{p}^{2}$ = 0.04] or on efficacy [*F*(2,61) = 0.30, *P* = 0.739, $\eta_{p}^{2}$ = 0.01]. However, there was a significant Group × Deck interaction on aversion [*F*(2,61) = 4.19, *P* = 0.020, $\eta_{p}^{2}$ = 0.12]. In probing the interaction within each participant group, patients with amygdala lesions did not perceive differences between the empathy and non-empathy deck [*t*(19) = −0.38, *P* = 0.705] but brain-damaged comparison participants [*t*(19) = 2.40, *P* = 0.027] and healthy comparison participants [*t*(23) = 4.14, *P* < 0.001] perceived the empathy deck as more aversive than the non-empathy deck.

In the cognitive EST, there were no significant Group × Deck interactions on effort [*F*(2,62) = 0.35, *P* = 0.703, $\eta_{p}^{2}$ = 0.01], on aversion [*F*(2,62) = 0.15, *P* = 0.859, $\eta_{p}^{2}$ = 0.00] or on efficacy [*F*(2,62) = 0.10, *P* = 0.902, $\eta_{p}^{2}$ = 0.00].

We additionally report separate Bayesian ANOVAs to determine the magnitude of the group difference effect size, 95% confidence region and report the region of practical equivalence (ROPE) using the bayesanova package in R (i.e. whether the magnitude of the effect size was no effect, small, medium or large and the highest percentage of support for the effect size).^50,51^ Bayesian comparisons are more elaboratively covered in Supplementary Table 2. Here, we summarize ratings on the empathy deck.

In the affective EST, for effort, comparisons revealed a small effect that patients with amygdala lesions rated empathy less effortful than brain-damaged comparison patients [posterior mean = −0.41, 95% CI (−0.64, −0.14), 76.61% posterior probability within ROPE of a small effect] and a medium effect against healthy comparison participants [posterior mean = −0.51, 95% CI (−0.70, −0.30), 56.83% posterior probability within ROPE of a medium effect]. For aversion, Bayesian comparisons revealed large effect sizes for patients with amygdala lesions against brain-damaged comparison patients [posterior mean = −1.22, 95% CI (−1.44, −0.96), 100% posterior probability within ROPE of a large effect] and against healthy comparison participants [posterior mean = −0.94, 95% CI (−1.13, −0.72), 91.91% posterior probability within ROPE of a large effect]. For efficacy, Bayesian comparisons revealed a small effect for patients with amygdala lesions against brain-damaged comparison patients [posterior mean = −0.49, 95% CI (−0.70, −0.25), 50.68% posterior probability within ROPE of a small effect] and a small effect against healthy comparison participants [posterior mean = −0.34, 95% CI (−0.55, −0.13), 89.24% posterior probability within ROPE of a small effect].

In summary, the strongest observation on cognitive costs for the affective EST was that patients with amygdala lesions rated the affective EST’s empathy deck as less aversive compared with the comparison groups given this was observed in the NHST and large effect size from Bayesian comparisons.

For the cognitive EST, when comparing patients with amygdala lesions against brain-damaged comparison patients or healthy comparison participants, Bayesian comparisons revealed across effort, aversion and efficacy, that more evidence supporting no effects was observed (Supplementary Table 2).

## Discussion

On two different free-choice tasks (i.e. the ESTs) examining motivation to engage in affective and cognitive forms of empathy, we obtained support for the conclusion that patients with amygdala lesions have preserved empathy motivation in relation to brain-damaged and healthy comparison groups. In exploratory analysis, we examined whether differences with engaging with empathy versus non-empathy deck instructions would explain any group differences or lack thereof. We found no notable differences in how the patients with amygdala lesions engaged with these tasks compared with the comparison groups in terms of their use of emotional words in the affective EST, nor did we find any group differences in the accuracy of emotional valence attributed to the self or others in the cognitive EST.

Additionally, other analyses found that patients with amygdala lesions showed no consistently strong differences from comparisons in how they perceived empathy—they perceived it as cognitively costly—although their ratings of affective empathy’s aversiveness in the affective EST were considerably lower than the ratings of the comparison groups (Table 4). It may be that amygdala dysfunction results in lower propensities for perceiving aversiveness in sharing other’s emotional experiences, given the strong evidence of approach bias in patients with amygdala lesions and emotion dysregulation.^17,18^ Alternatively, patients with amygdala lesions may have more of an emotional apathy, which may render the perception of trying to share in and understanding another person’s emotions and observing their external appearance seemingly equivalent.

In the Supplementary material, we report that patients with amygdala lesions did not differ from comparisons (those with non-amygdala lesions, as well as those with no lesions) in the extent to which their perceptions of the cognitive costs of empathy correlated with their proportion of choosing empathy decks (Supplementary Table 3).

We also report a fine-grained analysis looking at the proportion of damage to the amygdala in our participants in relationship to choosing empathy.^55,56^ Thus, we obtained no statistically significant evidence supporting the conclusion that the extent of damage to the amygdala, examined in a gradient manner, was associated with empathy motivation (Supplementary Table 4).

We also obtained individual differences and reports of DSM-5 facets (self and informant reports from PID-5; Supplementary Table 5)^47^ in addition to scores on the PPI-R (Supplementary Table 6)^49^ from our sample, and there were no significant differences between participant groups on these items or on individual differences on trait empathy measures (Supplementary Tables 7–9). Future research should consider adopting newer measures of empathy—such as the Questionnaire of Cognitive and Affective Empathy^57^—or social motivation—such as the Apathy Motivation Index^58^—which may help capture more nuanced impairments on empathy ability or motivation on a trait level.

These findings should provide caution to the views that amygdala dysfunction is directly linked with callous, sociopathic or psychopathic traits^4,5,59^ given our observations that these same patients did not replicate the strong patterns of empathy avoidance observed in healthy comparison participants in previous studies,^23,28,30^ nor did they avoid empathy differently than the comparison groups in our sample (Table 2). Plenty of research has documented links between amygdala dysfunction and empathic ability, such that even with comparable empathy motivation levels, patients with amygdala lesions may exhibit socially inappropriate or unexpected behaviour (e.g. issues of accurately recognizing negative emotions in others, exhibiting a lack of personal space around strangers and making inappropriate comments in front of new people).^18,60^ We juxtapose our research with this earlier work by emphasizing that our work specifically targeted empathy motivation, whereas previous research focused on empathic ability (e.g. accurately identifying the emotional states of other people). This earlier research has strongly documented that individuals with bilateral amygdala lesions displayed impairments with identifying fear and other social emotions in others.^2,61,62^ Our patients with amygdala lesions had unilateral lesions (13 left, 8 right) opposed to bilateral lesions. Previous research examining patients with unilateral amygdala lesions has found that these individuals perform comparably to brain-damaged and healthy comparison participants with recognizing social emotions (e.g. guilt, arrogance, admiration, flirtatiousness)^62,63^ or that those with right versus left amygdala damage (from temporal lobectomies) may be impaired on identifying fear from facial expressions.^64^ Ultimately, more research is needed to understand the extent of emotion recognition deficits for patients with unilateral amygdala lesions as they may differ from comparison groups depending on the type of emotion being recognized (e.g. social emotions, fear) as well as the laterality of damage (i.e. right versus left amygdala damage).

Nevertheless, these structures may have critical roles more broadly in the accuracy of empathy as documented previously (e.g. affective and cognitive theory of mind),^2,65,66^ but damage to these regions does not appear to substantively decrease motivation to empathize, at least as assessed in these two behavioural tasks. For instance, recent studies have documented that damage to the right amygdala was associated with improved mood after epilepsy surgery.^67^ Additionally, compared with trends observed in multiple larger samples using the EST in which people reliably avoided affective empathy^23^ and concern for others,^30^ patients with amygdala lesions exhibited no remarkable preference to avoid affective empathy compared with the neurologically healthy participants observed in previous research using the EST.^23,28,30^ Although notably, the healthy comparison individuals did not show preferences to avoid empathy either, with some implications for the individuals that comprise this sample compared with earlier research (i.e. older age).

Of course, the strength of such comparisons is limited by experimental design and demographic differences in the current study [including age of participants, testing location (i.e. remote versus in-person), and presence of multiple variants of EST within the same session]. Empathy often operates with multiple sub-facets, including experience sharing and perspective-taking, which we attempted to capture with the two EST variants here.^20,21,68^ The contribution of the amygdala may be implicated with one’s ability to accurately identify the emotional experiences of others.^5,69^ But the conclusion is clear: when we focused primarily on assessing motivation (compared with past studies which predominantly focused on ability), we found that patients with lesions to these regions are not necessarily impaired on empathy motivation.

As with most research with human lesion patients, the generalizability of our findings is limited by our sample size, characteristics and statistical power. A sensitivity analysis^70^ of our central question indicated we would have the ability to assess differences for the patients with amygdala lesions from comparison groups with an effect size *f* = 0.49 (medium). Nevertheless, group lesion studies have clinical value for studying changes in behaviour,^71^ are quite rare^72^ given the scarcity of patients with amygdala lesions and generally have larger effect sizes given the profound changes that occur following brain damage.^73^ Where appropriate, we supplement our primary NHST analyses with Bayesian frequentist comparisons to report whether considerable evidence for the null hypothesis was obtained. Additionally, our sample size was much larger than that of comparable lesion studies.^33,74^ We also used two EST variants (affective, cognitive) in order to capture motivation to experience empathy.^23,30^

It may also be that the task instructions for the FEEL deck in the affective EST provide an incrementally more complex series of steps that must be fulfilled (i.e. visually processing the person in addition to sharing their emotional experience) compared with the DESCRIBE deck (i.e. visually processing the person). Although we do not have specific data to support or refute this notion, earlier research pitted the choice of identifying the emotion on the face of targets in this type of task against additionally sharing the emotions to uncover that the emotional sharing component is less preferred, especially when perceived as cognitively costly.^23^ However, it is plausible that participants may have engaged both processes when choosing the FEEL (empathy) deck, and prior work has highlighted that empathy often operates with multiple processes operating together.^75^ Though this task may be somewhat removed from everyday experiences of empathy,^75^ it allowed us to isolate the experience of empathy and study people’s choices to empathize across multiple trials, as well as probe why they may or may not want to experience it.^76^ Thus, concerns of the EST lacking external validity may still be tolerated as long as the method can provide an internally valid test of the question of interest (i.e. here, an examination of empathy motivation in patients with amygdala lesions).^76,77^ Thus, we believe this test of empathic motivation provides a strong foundation for understanding whether patients with damage to the amygdala exhibit impairments in empathic motivation considering prior evidence of impaired ability.

Another possible confounding factor is the influence of impairments in emotion regulation on the EST. This task models situation selection, which may be an effective emotion regulation strategy for empathy.^78^ Arguably, Patient SM’s decision to approach rather than avoid pleas for help from complete strangers^14^ is a testament of the type of situation selection applied during empathy-relevant contexts in everyday life. If choosing to avoid empathy by picking the alternative deck involves downregulating empathic responses, then emotion regulation impairments, which sometimes accompany damage to the prefrontal cortex or amygdala,^79^ could increase the cognitive costs of that choice. More work is needed to better understand the extent to which any emotion regulation impairments may influence behaviour in the EST.^7,79,80^ Lastly, some of our patients with amygdala lesions additionally had lesions in adjacent structures which may also contribute to empathic functioning (e.g. medial temporal lobe, Fig. 1A).^81-83^ Although we supplement the group-based analyses with a proportion of amygdala damage analysis (Supplementary Table 4), being able to study those with more focal amygdala lesions may better highlight whether changes in motivation or ability influence overall empathy functioning.

Critically, this research highlights that motivation-based deficits need to be studied in addition to capacity-based deficits of empathy^19-21^ given both contribute to empathy’s success. Of course, capacity to empathize cannot be directly measured,^19^ and it may be that differences in opting into empathy-eliciting situations could complement differences in how far people can push their empathy once they are willing to do so. There may be a place for both motivation-based and capacity-based explanations of socioemotional deficits in these populations. This is noteworthy, given many theories link damage or dysfunction of the amygdala with highly stigmatized callous or psychopathic traits.^5,84^ However, more research is needed to compare this evidence against other longstanding evidence of their changes in personality, social behaviour and socioemotional function.^5^ For instance, previous work has suggested that psychopathic traits may be associated with a lack of motivation to empathize, but an intact ability to do so.^85^ Importantly, our findings indicate that patients with amygdala lesions may exhibit no remarkable impairments in empathy motivation, even though earlier research documented that these patients have compromised accuracy in affective and/or cognitive empathy,^2,6,11^ especially in complex social situations. Although these patterns of impairment might lead to similar behaviours, they differ fundamentally, and more research is needed to disentangle the two. Though earlier work found links with (right) amygdala volume and extraordinarily altruism (i.e. kidney donation),^4^ the inverse suggestion that people with lesions to the amygdala would show opposing characteristics requires careful investigation (i.e. in the case of Patient SM).^14^ Characterizing individuals with amygdala lesions as cold, uncaring or even callous could be stigmatizing people who are, in fact, comparably motivated to empathize with and help others.

## Conclusion

Individuals with lesions to the amygdala often exhibit impairments in empathy, emotion recognition and perspective-taking. This has led to several theories linking amygdala dysfunction and damage with the emergence of callous and psychopathic traits. However, we found that individuals with lesions to the amygdala did not display any exceptional impairments in their motivation to empathize with strangers and instead exhibit preserved empathic motivation. More research examining the neural basis of empathic motivation and the extent to which motivation might mediate capacity-based deficits in empathy will help clarify the role of this neural region to empathic functioning. It may be that the social deficits of individuals with damage to their amygdala, often interpreted as callousness, might reflect a miscalibration of their empathic abilities rather than a motivated indifference to the feelings of others.

## Supplementary Material

awag074_Supplementary_Data

## Data Availability

Data, syntax and analytic code are available (https://osf.io/d4epm).
