# Supplementary material for: Empathy motivation is preserved following amygdala damage
Source: Brain. 2026 Mar 13;149(5):1623–34. doi: 10.1093/brain/awag074 (PMC13140638; doi:10.1093/brain/awag074)
Supplement: awag074_Supplementary_Data [file awag074_supplementary_data.pdf]

Supplementary Material

Supplementary Hypotheses

We hypothesized that, across participants, perceiving empathy as more cognitively costly would be associated with choosing it less as documented in numerous previous studies using the EST (supplemental H3).[1–3] Lastly, we hypothesized that patients with amygdala lesions would have weaker correlations between perceiving empathy as cognitively costly and choosing it less than comparison groups (supplemental H4). These hypotheses were the same across the task variants. Exploratory analyses are also covered in Supplemental Material to examine the association between the proportion of amygdala damage with choosing empathy (given prior research documenting the necessity of the amygdala on empathy).[4]

Cognitive Costs Did Not Correlate with Empathy Motivation

We did not find consistent support for Supplemental H3 (Higher ratings of effort, aversiveness, and inefficacy on the empathy decks compared to the non-empathy decks within each task will be associated with reduced empathy choice). In the affective EST, when collapsing across groups there were no significant associations between choosing the empathy deck and reporting it more effortful (r = -0.05, p = .696, 95% CI [-0.29, 0.20]), aversive (r = -0.14, p = .277, 95% CI [-0.37, 0.11]), or efficacious (r = 0.20, p = .120, 95% CI [-0.05, 0.42]) than the non-empathy deck. In the cognitive EST, there was a negative association with choosing the empathy deck and rating it as more aversive than the non-empathy deck (r = -0.27, p = .028, 95% CI [-0.48, -0.03]) and a positive association with rating the empathy deck more efficacious than the non-empathy deck (r = 0.32, p = .010, 95% CI [0.08, 0.52], but no significant associations with rating it as more effortful (r = -0.12, p = .337, 95% CI [-0.35, 0.13]). However, it is important to note that our sample size is under-powered to detect individual difference correlations (i.e., for effect size f = .25 [medium] for a two-tailed correlation at 80% power and .05 alpha level, we would require 120 participants).[5]

The (non) association between perceived empathy costs and empathy avoidance did not differ for patients with amygdala lesions.

Lastly, we did not obtain support for Supplemental H4 (The amygdala lesion group will have lower correlations between proportion of choosing empathy and the relative perceived

effortfulness, aversiveness, and efficaciousness of empathy compared to non-empathy deck within each EST). We conducted comparisons of the associations of cognitive costs and empathy choice (see Supplemental Table 3) using the cocor package shiny web app.[6] The comparison test accounts for participant group sample size and correlation coefficients, and we conducted these at a .05 alpha level, 95% confidence interval, null value of 0, using two-tailed tests. None of the correlations between empathy choice and cognitive costs of the amygdala lesion group significantly differed from comparison groups.

Exploratory Analyses

Proportion of Amygdala Damage and Empathy Motivation

We examined whether the amount of damage to the amygdala was proportionally associated with choosing empathy in the experience sharing and mentalizing ESTs. That is to say, we examined whether individuals who were missing larger proportions of their left and/or right amygdala differed in their empathic choices. This provided another means of evaluating the contribution of these neural regions in motivation to empathize, by examining how the proportion of their loss was associated with empathy choice.[7,8] In these analyses, we excluded the healthy comparison participants given they did not have brain damage to examine, as well as one amygdala lesion and one brain-damaged comparison patient who did not have available mapped data. We found no statistically significant associations with the proportion of left or right amygdala damage with choosing the empathy deck more in the affective or cognitive EST (see Supplemental Table 4).

Affective EST Word Count Analyses

One could argue that the number of words written between the two decks (FEEL, DESCRIBE) in the affective EST might contribute to preferences to opt for the empathy versus non-empathy deck on this task. We explored this analysis and found that there was indeed a choice effect on the number of words written about the person in the image, such that participants were more likely to use more words on the empathy compared to the non-empathy deck choice trials ($b = 2.36$, $SE(b) = 0.54$, $p = < .001$). There was no group effect on the number of words used ($b = 0.37$, $SE(b) = 0.49$, $p = .458$). However, there was an interaction of deck choice and group on number of words used ($b = -0.63$, $SE(b) = 0.18$, $p < .001$).

When exploring the differences within each group, patients with amygdala lesions used more words on the empathy compared to non-empathy deck (b = 2.00, *SE*(b) = 0.38, $p < .001$). However, this was not the case for brain-damaged comparison patients (b = -0.26, *SE*(β) = 0.44, $p = .565$) or for healthy comparison participants (b = 0.20, *SE*(b) = 0.32, $p = .532$). This suggests that participants may be using more words on the empathy compared to non-empathy deck in the affective EST, but that these differences are mostly observed in patients with amygdala lesions.

In another exploratory analysis examining whether the ratio of words written on the FEEL versus DESCRIBE deck associated with choice proportions, when collapsing the analyses across all three groups, we do not obtain evidence that this ratio predicted the proportion of choosing empathy (FEEL) in that task, b = 0.00, *SE*(b) = 0.05, $t = -0.10$, $p = .920$. When running a crude split analyses within the three participant groups, there appears to be no strong evidence that writing more words in the empathy deck compared to non-empathy deck was associated with the proportion of choosing the empathy deck (amygdala: b = -0.12, *SE*(b) = 0.10, $p = .253$; brain-damaged control: b = 0.02, *SE*(b) = 0.06, $p = .775$; healthy comparison: b = 0.11, *SE*(b) = 0.09, $p = .228$). Therefore, this suggests that the number of words written on the EST is not a considerable influence on the choice patterns in the EST.

Supplemental Table 1 Pre-registration, Analytical, and Comparison Deviations

Hypothesis	Original	Deviation
H1	Neurological patients with focal amygdala lesions will choose empathy more compared to patients with focal vmPFC lesions, brain-damaged comparison patients, and demographically-matched healthy comparison participants.	Patients with amygdala lesions will choose empathy more than the comparison groups. [Comparisons for patients with vmPFC lesions is reported in a separate manuscript][9]
H2	Neurological patients with focal amygdala lesions will perceive less effort, aversion, and inefficacy with empathy, measured through the NASA Task Load Index, compared with patients who have focal vmPFC lesions, brain-damaged comparison patients, and demographically-matched healthy comparison participants.	Patients with amygdala lesions will perceive less cognitive cost associated with empathy compared to the comparison groups.
H3	Choosing empathy in the Empathy Selection Task will correlate with perceiving less effort, aversion, and inefficacy with empathy as measured through the NASA Task Load Index.	***No Deviation***.
H4 (or H3a)	Patients with focal amygdala lesions will have lower correlations of NASA Task Load Index effort, aversion, and inefficacy with empathy choice compared to patients with focal vmPFC lesions, brain-damaged comparison patients, and demographically-matched healthy comparison participants.	Patients with focal amygdala lesions will have lower correlations of NASA Task Load Index effort, aversion, and inefficacy with empathy choice compared to brain-damaged comparison patients and demographically-matched healthy comparison participants.
Analysis Approach	Amygdala (N = 21), vmPFC (N = 13), brain-damaged comparison (N = 35), healthy comparison (N = 40) with ANOVAs on group-effects.	Amygdala (N = 21), brain-damaged comparison (N = 22/35), healthy comparison (N = 24/40) with ANOVAs on group-effects and Bayesian frequentist analyses.
Exclusions	Participants with blank responses on either of the two Empathy Selection Tasks will be excluded from analyses of that particular Empathy Selection Task.	In the affective EST, one healthy comparison left three blank written responses (< 7.5%). In the cognitive EST, two amygdala lesion patients had missing valence ratings (≤ 5%). We retained these participants given they did not have missing choice data and had minimal missing responses.

Brain-damaged comparison and healthy comparison participants were matched with patients with amygdala and vmPFC lesion samples separately using the case-matching feature in SPSS (on age, sex, and education level).

Supplemental Table 2 Bayesian Comparisons of Cognitive Costs by Deck for Patients with Amygdala Lesions

				**Affective EST**					
	**Effort**			**Aversion**			**Efficacy**		
**FEEL**	$\bar{x}$	**95% CI**	**ROPE**	$\bar{x}$	**95% CI**	**ROPE**	$\bar{x}$	**95% CI**	**ROPE**
vs. BDC	-0.41	[-0.64, -0.14]	76.61% small	-1.22	[-1.44, -0.96]	100% large	-0.49	[-0.70, -0.25]	51.38% small
Vs. HC	-0.51	[-0.70, -0.30]	56.83% medium	-0.94	[-1.13, -0.72]	91.91% large	-0.34	[-0.54, -0.12]	89.98% small
**DESCRIBE**	$\bar{x}$	**95% CI**	**ROPE**	$\bar{x}$	**95% CI**	**ROPE**	$\bar{x}$	**95% CI**	**ROPE**
vs. BDC	-0.04	[-0.32, 0.14]	98.76% none	-0.36	[-0.55, -0.14]	90.63% small	-0.16	[-0.36, 0.04]	66.60% none
vs. HC	0.02	[-0.12, 0.16]	100% none	-0.05	[-0.21, 0.11]	99.56% none	-0.27	[-0.45, 0.06]	77.05% small
				**Cognitive EST**					
**FEEL-OTHER**	$\bar{x}$	**95% CI**	**ROPE**	$\bar{x}$	**95% CI**	**ROPE**	$\bar{x}$	**95% CI**	**ROPE**
vs. BDC	0.03	[-0.19, 0.24]	96.21% none	0.09	[-0.16, 0.33]	85.22% none	0.17	[-0.11, 0.44]	58.96% none
Vs. HC	-0.12	[-0.32, 0.10]	81.45% none	0.12	[-0.11, 0.35]	78.61% none	-0.20	[-0.42, 0.03]	51.13% none
**FEEL-SELF**	$\bar{x}$	**95% CI**	**ROPE**	$\bar{x}$	**95% CI**	**ROPE**	$\bar{x}$	**95% CI**	**ROPE**
vs. BDC	-0.17	[-0.32, -0.01]	66.03% none	-0.09	[-0.21, 0.03]	98.67% none	0.22	[-0.01, 0.42]	56.85% small
Vs. HC	-0.10	[-0.24, 0.05]	95.30% none	-0.04	[-0.16, 0.08]	100% none	-0.21	[-0.40, -0.02]	56.43% small

Supplemental Table 3 Correlations Between Cognitive Cost Difference Ratings and Empathy Choice

	**Amygdala Lesions Group**			
	versus Brain-Damaged Comparison		versus Healthy Comparison	
**Affective EST**	**Z**	***p***	**Z**	***p***
Effort	1.35	.177	0.65	.518
Aversiveness	1.54	.124	-0.37	.712
Efficacy	1.34	.180	0.63	.531
**Cognitive EST**	**Z**	***p***	**Z**	***p***
Effort	-0.21	.836	-1.10	.273
Aversiveness	1.07	.286	0.40	.686
Efficacy	0.88	.378	0.54	.587

Cognitive cost difference ratings are computed by the rating by subtracting the rating provided on the non-empathy inducing deck from the empathy-inducing deck (e.g., $Effort_{empathy}$ – $Effort_{non\text{-}empathy}$ = $EffortDifferenceRating_{effort}$).

Supplemental Table 4 Proportion of Amygdala Damage and Empathy Choice Correlations

	r	95% CI	*p*	*N*
**Affective EST**				
L amygdala	0.03	[-0.28, 0.34]	.854	40
R amygdala	0.06	[-0.25, 0.37]	.694	40
**Cognitive EST**				
L amygdala	0.09	[-0.23, 0.39]	.593	40
R amygdala	0.10	[-0.22, 0.39]	.555	40

Supplemental Table 5 DSM-5 Self and Informant Reports

	Amygdala Lesions					Brain-Damaged Comparison					Healthy Comparison				
**Latent Factor**	**Self**	**Other**	***t***	***p***	***N***	**Self**	**Other**	***t***	***p***	***N***	**Self**	**Other**	***t***	***p***	***N***
Negative Affect	0.94 (0.57)	0.68 (0.63)	1.16	.270	12	0.93 (0.55)	0.72 (0.45)	1.19	.259	12	0.70 (0.41)	0.53 (0.51)	1.87	.077	21
Detachment	0.69 (0.49)	0.57 (0.34)	1.20	.255	12	1.01 (0.55)	1.24 (0.76)	-1.28	.229	11	0.75 (0.49)	0.64 (0.58)	1.16	.260	21
Antagonism	0.51 (0.34)	0.13 (0.10)	4.23	.001	12	0.46 (0.40)	0.26 (0.26)	1.73	.115	11	0.58 (0.48)	0.34 (0.39)	2.65	.015	21
Disinhibition	0.58 (0.37)	0.56 (0.40)	0.15	.884	12	0.72 (0.34)	0.72 (0.49)	0.05	.959	12	0.43 (0.36)	0.31 (0.30)	1.42	.172	21
Psychoticism	0.45 (0.58)	0.29 (0.19)	1.00	.339	12	0.59 (0.46)	0.51 (0.32)	0.61	.554	12	0.42 (0.37)	0.30 (0.22)	1.65	.115	21

Participants completed the written self-report measures either in person or were given a take-home packet with the PID-5-IRF, PID-5, and PPI-R to complete and mail in. Participants were instructed to have a spouse, family member, roommate, or close friend who knew them well fill out the form and mail it in.

Supplemental Table 6 Psychopathic Personality Inventory Revised (PPI-R) Reports

	Amygdala Lesions	Brain-Damaged Comparison	Healthy Comparison	Group Effect		
**Item**	***M* (*SD*)**	***M* (*SD*)**	***M* (*SD*)**	***F***	***p***	η_p^2
Machiavellian Egocentricity	35.22 (6.73)	34.89 (7.80)	34.91 (7.04)	0.01	.988	0.00
Rebelliousness Nonconformity	26.89 (6.14)	29.78 (7.26)	25.57 (8.18)	1.70	.192	0.06
Blame Externalization	27.22 (9.18)	30.17 (9.78)	24.09 (5.02)	2.91	.063	0.09
Carefree Nonplanfulness	30.00 (6.69)	35.06 (4.07)	32.83 (7.78)	2.73	.074	0.09
Social Influence	42.61 (10.35)	39.22 (7.41)	43.09 (9.58)	0.99	.377	0.03
Fearlessness	27.44 (8.40)	30.72 (9.33)	24.52 (7.30)	2.83	.068	0.09
Stress Immunity	31.39 (7.95)	35.28 (6.75)	35.22 (7.11)	1.76	.182	0.06
Coldheartedness	28.44 (6.18)	29.67 (6.29)	33.35 (7.74)	2.89	.064	0.09
Virtuous Responding	29.78 (4.58)	29.28 (5.10)	31.26 (5.09)	0.91	.409	0.03
Deviant Responding	12.22 (1.77)	12.00 (2.00)	12.22 (3.20)	0.05	.951	0.00
**Total**	249.22 (25.00)	264.78 (30.47)	253.57 (39.36)	1.08	.345	0.04
**Self-Centered Impulsivity**	119.33 (19.76)	129.89 (21.44)	117.39 (20.69)	2.03	.140	0.07
**Fearless Dominance**	101.44 (18.74)	105.22 (18.45)	102.83 (19.57)	0.18	.833	0.01

There was data available for *N* = 18/21 in the amygdala lesion group, *N* = 18/22 in the brain-damaged comparison group, and *N* = 23/24 in the healthy comparison group.

Supplemental Table 7 Group Individual Differences

Individual Difference Measure	Amygdala Lesions *M* (*SD*)	BD Comparison *M* (*SD*)	Healthy Comparison *M* (*SD*)	*F*	*p*	η_p^2
IRI[a] - Fantasy	2.77 (0.76)	2.86 (1.07)	3.15 (0.75)	1.20	.308	0.04
IRI – EC[b]	3.91 (0.63)	3.69 (0.73)	3.92 (0.82)	0.66	.520	0.02
IRI – PT[c]	3.42 (0.83)	3.57 (0.62)	3.50 (0.62)	0.25	.782	0.01
IRI – PD[d]	2.41 (0.75)	2.24 (0.70)	2.30 (0.89)	0.26	.770	0.01
BAS[e] – Drive	2.63 (0.38)	2.63 (0.73)	2.54 (0.57)	0.16	.849	0.01
BAS – Fun Seeking	2.69 (0.48)	2.84 (0.55)	2.57 (0.53)	1.50	.232	0.05
BAS – Reward Responsiveness	3.35 (0.35)	3.29 (0.59)	3.38 (0.41)	0.23	.795	0.01
BIS[f]	2.95 (0.34)	2.95 (0.44)	3.09 (0.52)	0.72	.491	0.02
Self-Reported Altruism	2.04 (0.51)	2.08 (0.57)	1.98 (0.56)	0.17	.842	0.01

[a]IRI = Interpersonal Reactivity Index
[b]EC = Empathic Concern
[c]PT = Perspective Taking
[d]PD = Personal Distress
[e]BAS = Behavioral Activation System
[f]BIS – Behavioral Inhibition System
All measures were responded to on a 5-point scale. Due to a programming error, one item from the Self-Reported Altruism scale was not obtained from participants.

Supplemental Table 8 Correlations Between Individual Difference Measures and Empathy Choice

	Amygdala Lesions				BD Comparison				Healthy Comparison			
	r	*p*	95% CI	*N*	*r*	*p*	95% CI	*N*	*r*	*p*	95% CI	*N*
**Affective EST Choice Correlations**												
IRI[a] - Fantasy	0.19	.417	[-0.28, 0.58]	20	0.14	.544	[-0.31, 0.54]	21	0.28	.179	[-0.14, 0.61]	24
IRI – EC[b]	0.52	.018	[0.09, 0.78]	20	0.20	.392	[-0.26, 0.58]	21	0.09	.684	[-0.33, 0.47]	24
IRI – PT[c]	0.21	.380	[-0.26, 0.59]	20	0.51	.017	[0.09, 0.77]	21	0.36	.081	[-0.05, 0.66]	24
IRI – PD[d]	0.00	.991	[-0.44, 0.44]	20	-0.11	.621	[-0.52, 0.34]	21	-0.27	.203	[-0.60, 0.16]	24
BAS[e] – Drive	-0.29	.224	[-0.65, 0.19]	19	0.06	.794	[-0.38, 0.48]	21	0.00	.984	[-0.41, 0.40]	24
BAS – Fun Seeking	-0.20	.428	[-0.61, 0.30]	18	-0.06	.783	[-0.48, 0.38]	21	0.02	.935	[-0.39, 0.42]	24
BAS – Reward Responsiveness	0.07	.766	[-0.40, 0.51]	19	0.12	.595	[-0.33, 0.52]	21	0.09	.686	[-0.33, 0.47]	24
BIS[f]	0.00	.999	[-0.45, 0.45]	19	-0.15	.511	[-0.55, 0.30]	21	-0.28	.192	[-0.61, 0.15]	24
Self-Reported Altruism	0.42	.085	[-0.07, 0.73]	18	0.29	.209	[-0.17, 0.63]	21	0.13	.541	[-0.29, 0.51]	24
**Cognitive EST Choice Correlations**												
IRI - Fantasy	0.02	.926	[-0.44, 0.47]	19	-0.16	.489	[-0.54, 0.29]	22	0.05	.817	[-0.36, 0.44]	24
IRI – EC	0.08	.755	[-0.39, 0.51]	19	-0.13	.571	[-0.52, 0.31]	22	-0.08	.710	[-0.47, 0.34]	24
IRI – PT	-0.19	.446	[-0.59, 0.30]	19	-0.10	.670	[-0.50, 0.34]	22	-0.35	.097	[-0.65, 0.07]	24
IRI – PD	0.32	.182	[-0.17, 0.67]	19	-0.09	.690	[-0.49, 0.35]	22	0.03	.872	[-0.37, 0.43]	24
BAS – Drive	-0.41	.088	[-0.73, 0.08]	18	0.08	.718	[-0.35, 0.49]	22	0.24	.256	[-0.18, 0.58]	24
BAS – Fun Seeking	-0.41	.099	[-0.74, 0.10]	17	0.02	.919	[-0.40, 0.44]	22	0.19	.375	[-0.24, 0.55]	24
BAS – Reward Responsiveness	0.12	.622	[-0.37, 0.56]	18	-0.09	.698	[-0.49, 0.35]	22	0.06	.777	[-0.35, 0.45]	24
BIS	0.36	.142	[-0.14, 0.70]	18	-0.02	.929	[-0.44, 0.41]	22	-0.10	.628	[-0.49, 0.31]	24
Self-Reported Altruism	0.31	.228	[-0.21, 0.68]	17	0.10	.666	[-0.35, 0.51]	21	-0.14	.500	[-0.52, 0.28]	24

[a]IRI = Interpersonal Reactivity Index
[b]EC = Empathic Concern
[c]PT = Perspective Taking
[d]PD = Personal Distress
[e]BAS = Behavioral Activation System
[f]BIS – Behavioral Inhibition System

Supplemental Table 9 Correlation Comparisons Between Individual Difference Measures and Empathy Choice

	Amygdala Lesions Group			
	versus Brain-Damaged Comparison		versus Healthy Comparison	
**Affective EST**	**Z**	***p***	**Z**	***p***
IRI[a] - Fantasy	0.15	.879	-0.29	.770
IRI – EC[b]	1.10	.269	1.49	.136
IRI – PT[c]	-1.03	.301	-0.50	.616
IRI – PD[d]	0.33	.744	0.85	.396
BAS[e] – Drive	-1.04	.297	-0.90	.368
BAS – Fun Seeking	-0.41	.683	-0.66	.510
BAS – Reward Responsiveness	-0.15	.883	-0.06	.952
BIS[f]	0.44	.660	0.87	.386
Self-Reported Altruism	0.43	.670	0.94	.349
**Cognitive EST**	**Z**	***p***	**Z**	***p***
IRI - Fantasy	0.53	.593	-0.09	.928
IRI – EC	0.62	.534	0.48	.629
IRI – PT	-0.27	.786	0.52	.602
IRI – PD	1.24	.214	0.91	.363
BAS – Drive	-1.49	.135	-2.01	.044
BAS – Fun Seeking	-1.29	.196	-1.82	.069
BAS – Reward Responsiveness	0.61	.542	0.18	.858
BIS	1.15	.251	1.41	.158
Self-Reported Altruism	0.62	.537	1.34	.181

[a]IRI = Interpersonal Reactivity Index
[b]EC = Empathic Concern
[c]PT = Perspective Taking
[d]PD = Personal Distress
[e]BAS = Behavioral Activation System
[f]BIS – Behavioral Inhibition System

References

1. Cameron CD, Hutcherson CA, Ferguson AM, Scheffer JA, Hadjiandreou E, Inzlicht M. Empathy is hard work: People choose to avoid empathy because of its cognitive costs. *Journal of Experimental Psychology: General*. 2019;148(6):962-976.

2. Ferguson AM, Cameron CD, Inzlicht M. Motivational effects on empathic choices. *Journal of Experimental Social Psychology*. 2020;90:104010.

3. Scheffer JA, Cameron CD, Inzlicht M. Caring is costly: People avoid the cognitive work of compassion. *Journal of Experimental Psychology: General*. 2022;151(1):172-196. doi:10.1037/xge0001073

4. Lockwood PL, Cutler J, Drew D, et al. Human ventromedial prefrontal cortex is necessary for prosocial motivation. *Nature Human Behaviour*. Published online 2024:1-14.

5. Faul F, Erdfelder E, Buchner A, Lang AG. Statistical power analyses using G* Power 3.1: Tests for correlation and regression analyses. *Behavior research methods*. 2009;41(4):1149-1160.

6. Diedenhofen B, Musch J. cocor: A Comprehensive Solution for the Statistical Comparison of Correlations. Olivier J, ed. *PLoS ONE*. 2015;10(4):e0121945. doi:10.1371/journal.pone.0121945

7. Baas D, Aleman A, Kahn RS. Lateralization of amygdala activation: a systematic review of functional neuroimaging studies. *Brain Research Reviews*. 2004;45(2):96-103.

8. Lashley KS. In search of the engram. Published online 1950. Accessed April 28, 2024. https://psycnet.apa.org/record/1952-05966-020

9. Scheffer JA, Reber J, Cameron CD, Tranel D. Is damage to the ventromedial prefrontal cortex associated with impaired empathy? A neuropsychological study using an empathy motivation task. *2025*.
